# Comparative effectiveness of different consolidation chemotherapy regimens for pediatric acute lymphoblastic leukemia: A protocol for systematic review and network meta analysis

**DOI:** 10.1097/MD.0000000000022208

**Published:** 2020-09-18

**Authors:** Guoming Chen, Ruilan Huang, Zhuoxin Huang, Ziyin Chen, Huiping Liu, Jinfeng Wu, Zhiqiang Chen, Tianqi Gao, Hua Xu, Hai Lan

**Affiliations:** a Guangzhou University of Chinese Medicine; b First Affiliated Hospital of Guangzhou University of Chinese Medicine, Guangzhou; c Shunde Hospital of Guangzhou University of Chinese Medicine, Foshan, China.

**Keywords:** consolidation chemotherapy, network analysis, pediatric acute lymphoblastic leukemia, protocol

## Abstract

**Background:**

Acute lymphoblastic leukemia (ALL) is one of the most commonly seen cancers in children, which mainly relates with inherited genetic variations. Consolidation chemotherapy is usually given to the pediatric ALL patients, however there is no meta-analysis and network analysis conducting the efficacy of the chemotherapy. Therefore, we perform a protocol to assess the efficacy of chemotherapeutics for pediatric ALL.

**Methods:**

A literature search for randomized controlled trials about some specific chemotherapy regimens for pediatric ALL will be carried out in 7 electronic databases from their establishment to June 2019: the Cochrane Library, Embase, MEDLINE, the Chinese National Knowledge Infrastructure (CNKI), the Sino Med, the Chinese Scientific Journal Database (VIP) and the Wanfang Database. Complete continuous remission will be measured as primary outcome. Stata 14.0 will be utilized to perform a standard pairwise meta-analysis and the NMA, as well as draw Network Plots of Network Meta.

**Results:**

This network meta-analysis will evaluate the efficacy of different consolidation chemotherapy regimens.

**Conclusion:**

This study will furnish decision-making reference on optimum proposal of chemotherapy regimens for pediatric ALL.

**PROSPERO registration number:**

PROSPERO CRD42019134518

## 1 Introduction

Acute lymphoblastic leukemia (ALL), one of the most common malignancies, is diagnosed approximately 4000 cases in the USA, which is predominantly happen in children and adolescent.^[1]^ The onset of ALL is mainly because of inherited genetic variations, exogenous, or endogenous exposures and chances may also be the pathogenic factors.^[2,3]^ Treatment for ALL is based on its genotype, phenotype, and risk for its heterogeneity. Except for mature B-cell ALL treating by short-term intensive chemotherapy, other types’ specific treatments are different but in the routine that giving the remission-induction therapy at first and continuation treatment following consolidation therapy for reducing residual disease.^[4,5]^ CNS precaution and treatment and allogeneic hemopoietic cell transplantation will be used based on whether the patient is high risk or not.^[6]^ Relapsed ALL and second neoplasms are the main causes of death in ALL survivors.^[7,8]^

With the development of therapy for childhood ALL, the 5-year survival has been increased from 83.7% in early 1990s to 90.4% in 20th century, infants, however, still remain in a low rate.^[9]^

Consolidation chemotherapy is given after remission-induction therapy. The most popular used treatment protocol is vincristine, dexamethasone, asparaginase, with or without anthracycline.

Vincristine and asparaginase combined with prednisolone could make a positive role on the ALL patients, but probably increased the early event risk on resistant patients.^[10]^ When using vincristine and prednisone, ALL patients’ event-free survival was improved.^[11]^ In a study, the ALL patients received asparaginase greater than 26 weeks had a better outcome than those who tolerated 25 weeks or fewer, which means the effect of asparaginase may have a positive correlation with using duration.^[12]^ However, different studies enrolled different sample, and research design are in difference either. Therefore, different chemotherapeutics could not be compared directly so that the efficacy is uncertain.

Network meta-analysis which includes all the eligible randomized controlled trials will be performed to collect the statistical data, analyze the efficiency of different chemotherapeutics and find out the best regimen. The purpose of this network analysis is to compare the efficacy of different chemotherapeutics in the treatment for children diagnosed ALL.

## 2 Methods

This NMA protocol has been formulated in accordance with the Preferred Reporting Item for Systematic Reviews and Meta-Analysis Protocols (PRISMA-P) checklist and registered under PROSPERO (CRD 42019134518).

### 2.1 Ethics approval and dissemination

Given that this study will be carried out without patient involvement, no research ethical issue is required. The results of this research will be published in a peer-reviewed journal.

### 2.2 Criteria for considering studies for this review

#### 2.2.1 Types of included studies

Only randomized controlled trials in Chinese or English will be recruited and no publication date or publication status restrictions will be initially applied. Animal experiments, case reports, review papers, human cell or tissue experiments will be unavailable for this study.

#### 2.2.2 Types of participants

Enrolled patients (aged 1–21 years, regardless of gender, ethnicity, nationality or duration of disease) were clearly diagnosed with ALL.

#### 2.2.3 Types of interventions

Based on the literature, therapies for ALL are of wide variation including: Vincristine,6-Mercaptopurine, dexamethasone, methotrexate, and low dose of asparaginase(VDMALD); Vincristine,6-Mercaptopurine, prednisone, methotrexate, doxorubicin and arabinoside (VPMDA), or VPMDA regimen plus high dose of asparaginase(VPDMAAHD);Vincristine,6-Mercaptopurine, prednisone, methotrexate, and daunorubicin (VPMD);Vincristine,6-Mercaptopurine, prednisone and daunorubicin with low dose of methotrexate (VPDMLD); or VPMD regimen plus asparaginase and arabinoside (VPMDAA); and VPMDAA regimen with high dose of methotrexate (VPDAAMHD);sequential or alternating chemotherapy regimens with VPMDAA as the footstone (VPMDAAescalated). Studies assessing the efficacy and safety of any of the treatments listed above will be enrolled.

#### 2.2.4 Types of outcome measures

Primary outcome indicator will be complete continuous remission, which is defined as a crucial part of disease prognosis and treatment assessment of ALL.

### 2.3 Search methods for the identification of studies

Seven electronic databases including 4 Chinese databases (CNKI, SinoMed, Wanfang Database, and the Chinese Scientific Journal Database [VIP]) and 3 English databases (MEDLINE, Cochrane Library, and Embase) will be exhaustively and systematically searched from inception to June 2019. In addition, relevant data will be augmented to complete the deficiencies of the electronic databases through hand searching a range of relevant websites and checking the reference lists. The preliminary and validated retrieval strategy of PubMed is performed in Table 1, which will be restructured in conformity to specific databases.

**Table 1:**
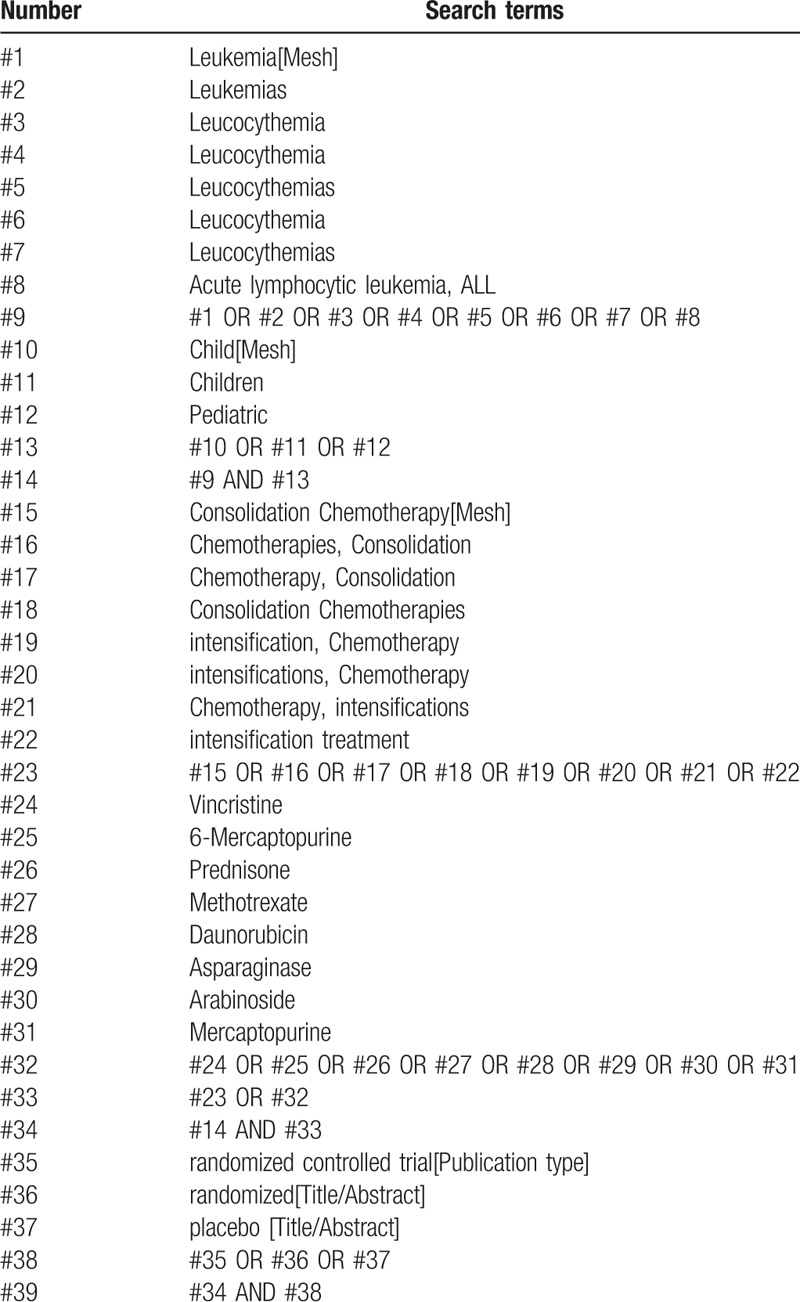
Search strategy for the PubMed database.

### 2.4 Study selection

**Figure 1:**
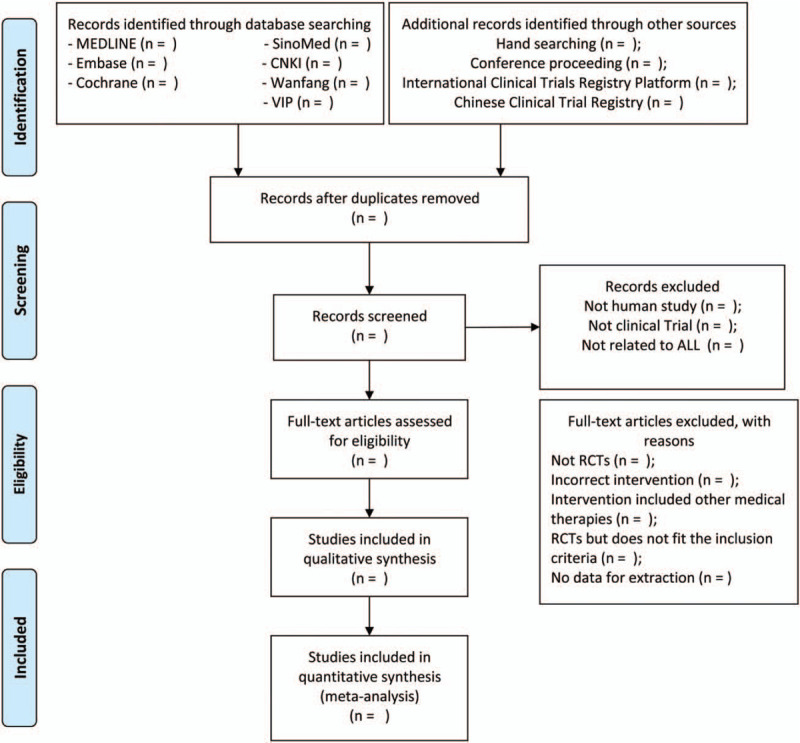
PRISMA flow chart of study selection process.

The two evaluation staffs (ZC and HL) will select the articles independently according to the titles and abstracts after combining all the qualified documents and eliminating duplicates using the EndNote X9. Subsequently, the remaining articles will be subjected to a full-text review for identification according to the prespecified criteria. Inconsistencies will be addressed by the discussion with a third reviewer (GC). The process of the study selection is presented in a Preferred Reporting Item for Systematic Reviews and Meta-Analysis (PRISMA) flow diagram (Fig. 1)

### 2.5 Data extraction

Data will be entered into the predefined spreadsheet implemented in Microsoft Excel and checked for accuracy by 2 independent reviewers (ZC and HL). Data items include publication information, characteristics of trial subjects, interventions, and outcomes. Any divergence will be settled by consensus or discussion with the third reviewer (GC). Whenever necessary, we will make an attempt to contact the authors for clarification of any confounding factors. If no response is received, the study will be discarded.

### 2.6 Quality evaluation on methodology

The methodological quality in individual studies will be assessed using Cochrane Review Handbook ^[13]^ independently by 2 verifiers (RH and ZH). Across the 7 domains (random sequence, allocation concealment, blinding of participants, and personnel, allocation concealment, incomplete outcome data, selective reporting, and other bias and risk). If needed, the third author (GC) will act as an arbitrator in case of discrepancy.

### 2.7 Statistic analysis

#### 2.7.1 Pairwise meta-analysis

Stata 14.0 software will be used for a standard pairwise meta-analysis. Mean difference will be selected as the effect size expressions for continuous variables while the dichotomous outcomes will be expressed as the odds ratio (OR) with associated 95% CIs.

We will perform a quantitative analysis of heterogeneity variances for each trial by *χ*^2^ test and *I*^2^ test. Considerable heterogeneity will exist among enrolled trials when *I*^2^ > 50% and *P* < .1, for which a random effects model would be chosen; otherwise the Mantel-Haenszel fixed model will be applied.

If there is heterogeneity between the results, subgroup analysis or meta-regression would be envisaged to carried out to investigate probable sources of heterogeneity.

We will conduct sensitivity analysis to ascertain the data reliability and seek to detect and analyze the abnormal study leading to the substantial heterogeneity. Funnel plot will be examined to evaluate publication bias which might cut down the evidence intensity.

#### 2.7.2 Network meta-analysis

STATA 14.0 will be utilized to perform the NMA as well as draw Network Plots of Network Meta.

If there is a closed loop, inconsistency factor will be applied to estimate (with 95% CIs) heterogeneity of enrolled trails.

Surface under the cumulative ranking will be utilized to evaluate the underlying ranking probability of interventions. The higher surface under the cumulative ranking value stands for better efficacy.

We will carry out a comparison-adjusted funnel plot to appraisal the existence of small-study effect.

### 2.8 Quality of evidence

The rate of all the inclusive literature will be assessed with the GRADE system with the designated grades of high quality, moderate quality, low quality, and very low quality. The certainty of evidence will be downgraded based on the follows: limitations in the design, unaccounted heterogeneity, unconformity, indirectness of evidence, hidden error, and high possibility of publication bias.

### 2.9 Patient and public involvement

No patients or public were involved.

## 3 Discussion

Until now, VPMD regimen is regarded as first-line drugs for pediatric ALL owing to its better curative effect and fewer untoward effect. However, the recognition and researches of asparaginase for intensification have got some new progressions for the past few years. Another way to improve outcomes is using high-dose Ara-C or MTX in the postremission or induction phase.^[14,15]^ At present, there is still lack of the normalized chemotherapy for ALL and the dosage in consolidation therapy has not reached a consensus yet. So the purpose of this study is to perform a network meta-analysis to comprehensively appraise the interests of different consolidation chemotherapy regimens.

Nevertheless, our study is somewhat limited in some factors. The authors expect to refer only English and Chinese literature, which may issue in the potential risk of omitting essential literature. And some literature of low quality may impact on the final results of this NMA. However, it is our hope that this study will conduce to future clinical trials and study design.

## Author contributions

**Conceptualization:** Zhiqiang Chen, Tianqi Gao.

**Data curation:** Zhuoxin Huang, Ziyin Chen, Huiping Liu, Jinfeng Wu.

**Funding acquisition:** Hai Lan.

**Investigation:** Ziyin Chen, Huiping Liu, Zhiqiang Chen, Tianqi Gao.

**Methodology:** Ruilan Huang, Zhuoxin Huang.

**Project administration:** Tianqi Gao.

**Resources:** Ziyin Chen, Huiping Liu.

**Software:** Jinfeng Wu.

**Supervision:** Hua Xu, Hai Lan.

**Writing – original draft:** Guoming Chen, Ruilan Huang.

**Writing – review & editing:** Guoming Chen.

## Abbreviations

Abbreviations: ALL = acute lymphoblastic leukemia, VPMD = Vincristine, 6-Mercaptopurine, prednisone, methotrexate and daunorubicin, VPMDAA = VPMD regimen plus asparaginase and arabinoside.

## References

[1] Pui CH Mullighan CG Evans WE. Pediatric acute lymphoblastic leukemia: where are we going and how do we get there? Blood 2012;120:1165 74. 22730540 10.1182/blood-2012-05-378943 PMC3418713

[2] Inaba H Greaves M Mullighan CG. Acute lymphoblastic leukaemia. Lancet 2013;381:1943 55. 23523389 10.1016/S0140-6736(12)62187-4 PMC3816716

[3] Moriyama T Relling MV Yang JJ. Inherited genetic variation in childhood acute lymphoblastic leukemia. Blood 2015;125:3988 95. 25999454 10.1182/blood-2014-12-580001 PMC4481591

[4] Pui CH Campana D Pei D. Treating childhood acute lymphoblastic leukemia without cranial irradiation. N Engl J Med 2009;360:2730 41. 19553647 10.1056/NEJMoa0900386 PMC2754320

[5] Pui CH Evans WE. Treatment of acute lymphoblastic leukemia. N Engl J Med 2006;354:166 78. 16407512 10.1056/NEJMra052603

[6] Florent M Mohamad M. Acute lymphoblastic leukaemia. Lancet (London, England) 2020;395:1146 62. 32247396 10.1016/S0140-6736(19)33018-1

[7] Mody R Li S Dover DC. Twenty-five-year follow-up among survivors of childhood acute lymphoblastic leukemia: a report from the Childhood Cancer Survivor Study. Blood 2008;111:5515 23. 18334672 10.1182/blood-2007-10-117150 PMC2424150

[8] MacArthur AC Spinelli JJ Rogers PC. Mortality among 5-year survivors of cancer diagnosed during childhood or adolescence in British Columbia, Canada. Pediatr Blood Cancer 2007;48:460 7. 16767717 10.1002/pbc.20922

[9] Hunger SP Lu X Devidas M. Improved survival for children and adolescents with acute lymphoblastic leukemia between 1990 and 2005: a report from the children's oncology group. J Clin Oncol 2012;30:1663 9. 22412151 10.1200/JCO.2011.37.8018 PMC3383113

[10] Den Boer ML Harms DO Pieters R. Patient stratification based on prednisolone-vincristine-asparaginase resistance profiles in children with acute lymphoblastic leukemia. J Clin Oncol 2003;21:3262 8. 12947061 10.1200/JCO.2003.11.031

[11] Lange BJ Bostrom BC Cherlow JM. Double-delayed intensification improves event-free survival for children with intermediate-risk acute lymphoblastic leukemia: a report from the Children's Cancer Group. Blood 2002;99:825 33. 11806983 10.1182/blood.v99.3.825

[12] Silverman LB Gelber RD Dalton VK. Improved outcome for children with acute lymphoblastic leukemia: results of Dana-Farber Consortium Protocol 91-01. Blood 2001;97:1211 8. 11222362 10.1182/blood.v97.5.1211

[13] Higgins JPT Thomas J Chandler J. *Cochrane Handbook for Systematic Reviews of Interventions version 6.0* (updated July 2019). Cochrane 2019. Available at: www.training.cochrane.org/handbook. Accessed June 30, 2020.

[14] Stevens RF Hann IM Wheatley K. Marked improvements in outcome with chemotherapy alone in paediatric acute myeloid leukemia: results of the United Kingdom Medical Research Council's 10th AML trial. MRC Childhood Leukaemia Working Party. Br J Haematol 1998;101:130 40. 9576193 10.1046/j.1365-2141.1998.00677.x

[15] Wells RJ Woods WG Lampkin BC. Impact of high-dose cytarabine and asparaginase intensification on childhood acute myeloid leukemia: a report from the Childrens Cancer Group. J Clin Oncol 1993;11:538 45. 8445429 10.1200/JCO.1993.11.3.538

